# Altered Arterial Stiffness and Subendocardial Viability Ratio in Young Healthy Light Smokers after Acute Exercise

**DOI:** 10.1371/journal.pone.0026151

**Published:** 2011-10-10

**Authors:** Robert J. Doonan, Patrick Scheffler, Alice Yu, Giordano Egiziano, Andrew Mutter, Simon Bacon, Franco Carli, Marios E. Daskalopoulos, Stella S. Daskalopoulou

**Affiliations:** 1 Department of Medicine, McGill University, Montreal, Quebec, Canada; 2 Department of Exercise Science, Concordia University, Montreal, Quebec, Canada; 3 Montreal Behavioural Medicine Centre, Hopital du Sacre-Coeur de Montreal, Montreal, Quebec, Canada; 4 Research Centre, Montreal Heart Institute, Montreal, Quebec, Canada; 5 Department of Anesthesia, Faculty of Medicine, McGill University, Montreal, Quebec, Canada; 6 Department of Vascular Surgery, Athens University, Athens, Greece; Virginia Commonwealth University, United States of America

## Abstract

**Background:**

Studies showed that long-standing smokers have stiffer arteries at rest. However, the effect of smoking on the ability of the vascular system to respond to increased demands (physical stress) has not been studied. The purpose of this study was to estimate the effect of smoking on arterial stiffness and subendocardial viability ratio, at rest and after acute exercise in young healthy individuals.

**Methods/Results:**

Healthy light smokers (n = 24, pack-years = 2.9) and non-smokers (n = 53) underwent pulse wave analysis and carotid-femoral pulse wave velocity measurements at rest, and 2, 5, 10, and 15 minutes following an exercise test to exhaustion. Smokers were tested, 1) after 12h abstinence from smoking (chronic condition) and 2) immediately after smoking one cigarette (acute condition). At rest, chronic smokers had higher augmentation index and lower aortic pulse pressure than non-smokers, while subendocardial viability ratio was not significantly different. Acute smoking increased resting augmentation index and decreased subendocardial viability ratio compared with non-smokers, and decreased subendocardial viability ratio compared with the chronic condition. After exercise, subendocardial viability ratio was lower, and augmentation index and aortic pulse pressure were higher in non-smokers than smokers in the chronic and acute conditions. cfPWV rate of recovery of was greater in non-smokers than chronic smokers after exercise. Non-smokers were also able to achieve higher workloads than smokers in both conditions.

**Conclusion:**

Chronic and acute smoking appears to diminish the vascular response to physical stress. This can be seen as an impaired ‘vascular reserve’ or a blunted ability of the blood vessels to accommodate the changes required to achieve higher workloads. These changes were noted before changes in arterial stiffness or subendocardial viability ratio occurred at rest. Even light smoking in young healthy individuals appears to have harmful effects on vascular function, affecting the ability of the vascular bed to respond to increased demands.

## Introduction

Arterial stiffness is considered a composite measure of vascular health and a predictor of cardiovascular events independent of traditional risk factors; it is caused by structural changes in the vascular wall, including fibrosis, medial smooth muscle cell necrosis, breaks in elastin fibers, calcifications, and diffusion of macromolecules into the arterial wall[1]–[3]. Notably, in the Framingham Heart Study, a one standard deviation (SD) increment in arterial stiffness, as measured by carotid-femoral pulse wave velocity (cfPWV), the ‘gold standard’, was associated with a 48% increase in arterial disease risk, independently of individual vascular risk factors[3]. Furthermore, a meta-analysis found that an increase in cfPWV by 1 m/s corresponded to an age-, sex-, and risk factor-adjusted risk increase of 14%, 15%, and 15% in total cardiovascular (CV) events, CV mortality, and all-cause mortality, respectively[2]. An increase in cfPWV by 1 SD was associated with respective increases of 47%, 47%, and 42%[2].

Using applanation tonometry, pulse wave analysis (PWA) and PWV measurements can be performed. PWA can provide important information about several arterial stiffness and hemodynamic parameters including augmentation index (AIx) and subendocardial viability ratio (SEVR), an indicator of myocardial workload and perfusion (O_2_ supply vs. demand)[4], [5]. Low SEVR has been shown to be associated with coronary artery disease, decreased coronary flow reserve in patients with healthy coronary arteries, severity of type I and type II diabetes, decreased renal function, and a history of smoking[4]–[13].

Cigarette smoking is the largest preventable cause of cardiovascular death and disability around the world[14]–[16]. While in general smoking rates have been declining in recent years, they remain quite high especially in young populations; 26.5% of people aged 20–34 years smoke in Canada and 21.8% of people aged 18–24 smoke in the Unites States[17], [18]. These figures are disturbing considering the detrimental impact of smoking. More recently smoking has been shown to increase arterial stiffness both acutely and chronically[19]. However, to date, studies have investigated this effect only on *resting* arterial stiffness. Nevertheless, people do not spend their lives at rest, and a good part of the day is spent doing short bouts of physical activity, some of which are vigorous (i.e. climbing stairs), producing a physical stress on the vascular system.

It is conceivable that examination of the response of the arteries to physical stress, such as acute intense exercise, could offer additional critical information about vascular health. In some individuals acute physical stress can reveal cardiovascular abnormalities that are not present at rest. Quantification of hemodynamic changes [e.g. PWA, SEVR, PWV parameters] that occur as the arterial system accommodates for the changes in heart rate, cardiac output, and blood flow (termed herein *‘arterial stress test’*) allows for comparisons between individuals and over time within the same person.

The purpose of this study was to estimate the extent to which chronic (study A) and acute (study B) smoking compromise arterial stiffness and SEVR at rest and after physical stress in young healthy individuals. To date, no published studies have assessed the effect of smoking on arterial stiffness after physical stress. This is particularly important considering that problematic vascular responses might be revealed only under physical stress.

## Methods

This study was approved by the ethics and scientific reviews boards of the McGill University Health Centre. Written informed consent was obtained for all participants.

### Participants

Using local university advertisements and recruitment within the McGill University Health Centre, consecutive young healthy male light smokers (n = 24) and non-smokers (n = 53), were enrolled in the study. Light smoking has variable definitions, being previously classified as high as ≤20 cigarettes per day[20]. Based on previous studies which have examined the effect of smoking on vessel hemodynamics in young healthy subjects with a mean of 10-16-pack years[21], [22], we have set the limit to smoking ≤15 cigarettes/day with ≤8 pack-year smoking history in order to obtain a population with lighter smoking history. Exclusion criteria were: previously diagnosed cardiovascular disease, traditional cardiovascular risk factors, renal disease, inflammatory diseases, obesity (body mass index, BMI ≥ 30 kg/m^2^), and/or any conditions/diseases that could affect arterial stiffness and/or exercise capacity. Furthermore, participants who were on regular cardioprotective medications or were acutely ill were not eligible to participate in this study.

### Study Design

We performed two studies: study A assessing the chronic effect and study B assessing the acute effect of smoking on arterial stiffness and SEVR. Non-smokers performed the arterial stress test protocol once (see below). For study A (chronic study) smokers performed the protocol after 12h abstinence from smoking and for study B (acute study) smokers abstained from smoking for 12h and then smoked 1 cigarette immediately before performing the protocol. Studies A and B were performed in a randomized fashion order. For the acute smoking study, smokers were asked to smoke 1 full standardized cigarette (nicotine content: 1.1–2.4 mg) within 5 minutes. All measurements were performed in the morning.

### Arterial Stiffness and Hemodynamic Measurements

Blood pressure was measured using cuff sphygmomanometry (HEM-705CP, Omron Corp., St. Charles, Illinois, United states) according to the Canadian Hypertension Education Program guidelines[23]. PWA, SEVR, and PWV measurements were performed using applanation tonometry (SphygmoCor, AtCor Medical, Sydney, Australia). For PWA, an average radial pressure waveform was generated from 10 sec of sequential radial pressure waveforms. Using a previously validated generalized transfer function, the system software calculated an averaged radial artery waveform (calibrated with brachial systolic and diastolic pressure) and derived a corresponding aortic pressure waveform [as well as the aortic pressure and the augmentation index adjusted to a heart rate of 75 (AIx75)][24], [25]. PWA was also used to determine SEVR [area under the curve (AUC) during diastole/AUC during systole]. cfPWV was measured using applanation tonometry and a 3-lead electrocardiogram. The validity of the derived aortic pressures and arterial waveforms has been confirmed by simultaneously recorded direct arterial measurements (catheterization) in a large number of participants of different ages and with different blood pressures at rest, during, and after exercise (r = 0.995, P<0.001)[25]–[29]. The SphygmoCor system used herein is highly reproducible (inter- and intra-operator) in both healthy and diseased populations[30], [31].

Information about medical history, current smoking status, and smoking history were directly queried. Pack-years were calculated; number of pack-years  =  (packs smoked per day) x (years as a smoker) (1 pack is considered as 20 cigarettes). Height, weight, and waist and hip circumference were recorded.

### Arterial Stress Test Protocol

Prior to undergoing the arterial stress test, participants were asked to abstain from: i) caffeine and ethanol intake for at least 12 h and ii) any strenuous exercise (aerobic or anaerobic) for 24 h. After 10 minutes of rest in a supine position in a temperature and humidity controlled environment, brachial blood pressure, PWA, and cfPWV measurements were performed in duplicate. The points of measurements were marked to ensure that measurements were performed on the same spots post-exercise. To induce physical stress, participants subsequently completed a supervised incremental treadmill exercise protocol to volitional exhaustion (Bruce protocol[32]), which has been validated in young healthy individuals[32]; throughout the test heart rate was monitored. Participants were deemed to reach maximal exercise capacity when all three of the following criteria were met: i) the participant could no longer continue the exercise protocol, ii) a minimum of 19 was reached on the Borg scale[32], and iii) the participant reached at least 90% of their age-predicted maximum heart rate. Time to exercise completion was recorded. Immediately post-exercise, participants rested in a supine position. At 2 minutes post-exercise, cfPWV was assessed once, and at 5, 10, and 15 minutes post-exercise cfPWV and PWA measurements were each performed once, in that order. Brachial blood pressure was measured at the same time points as arterial stiffness measurements in the contralateral arm, after confirmation of absence of difference in blood pressure between the two arms at rest (<5/3mmHg for systolic and diastolic blood pressure)[33]. All measurements were in accordance with the Sphygmocor internal quality control system. The above mentioned protocol constitutes the *‘arterial stress test’*.

### Statistical Analysis

SAS version 9.2 software (SAS Institute, 100 SAS Campus Dr, Cary, NC) was used for all statistical analyses. Demographic characteristics of smokers and non-smokers were compared on age, BMI, and waist:hip circumfrence using independent t-tests. Between-group comparisons (non-smokers and smokers) of resting parameters were performed using general linear models without and with adjustment for age and resting mean arterial pressure (MAP) (except MAP and aortic pulse pressure (PP)) using analysis of covariance (ANCOVA). Aortic PP and MAP comparisons were adjusted for age only. Post-exercise and peak change ( = greatest post-exercise value minus resting value) between-group comparisons for all parameters except for MAP and aortic PP were performed in a similar manner but with adjustment for age, resting MAP, exercise time, and the resting value of the parameter. Aortic PP and MAP were adjusted for age, exercise time, and resting aortic PP and resting MAP, respectively. General linear model repeated measures analysis of variance was used to assess differences in resting values (with adjustment for MAP) and post-exercise (with adjustment for exercise time and resting MAP) in smokers between the chronic condition and acute smoking condition.

To estimate the impact of smoking on overall vascular function after physical stress (acute exercise), AUC was calculated from the resting value for each participant for PWA and PWV measurements[34]. This method provides an estimate of overall vascular function, a continuous construct that can be measured at discrete time intervals only, while taking into consideration variations in AUC that may have occurred due to different values at rest. Differences in the AUC between non-smokers and smokers in each different condition were estimated using linear regression adjusting for age, resting MAP, and maximal exercise time with ANCOVA. AUC of arterial stiffness parameters were considered the dependent variables. General linear model repeated measures analysis of variance was used to assess differences in AUCs in smokers between the chronic condition and acute smoking conditions with adjustment for exercise time and resting MAP.

## Results

### Participant characteristics

**Figure 1:**
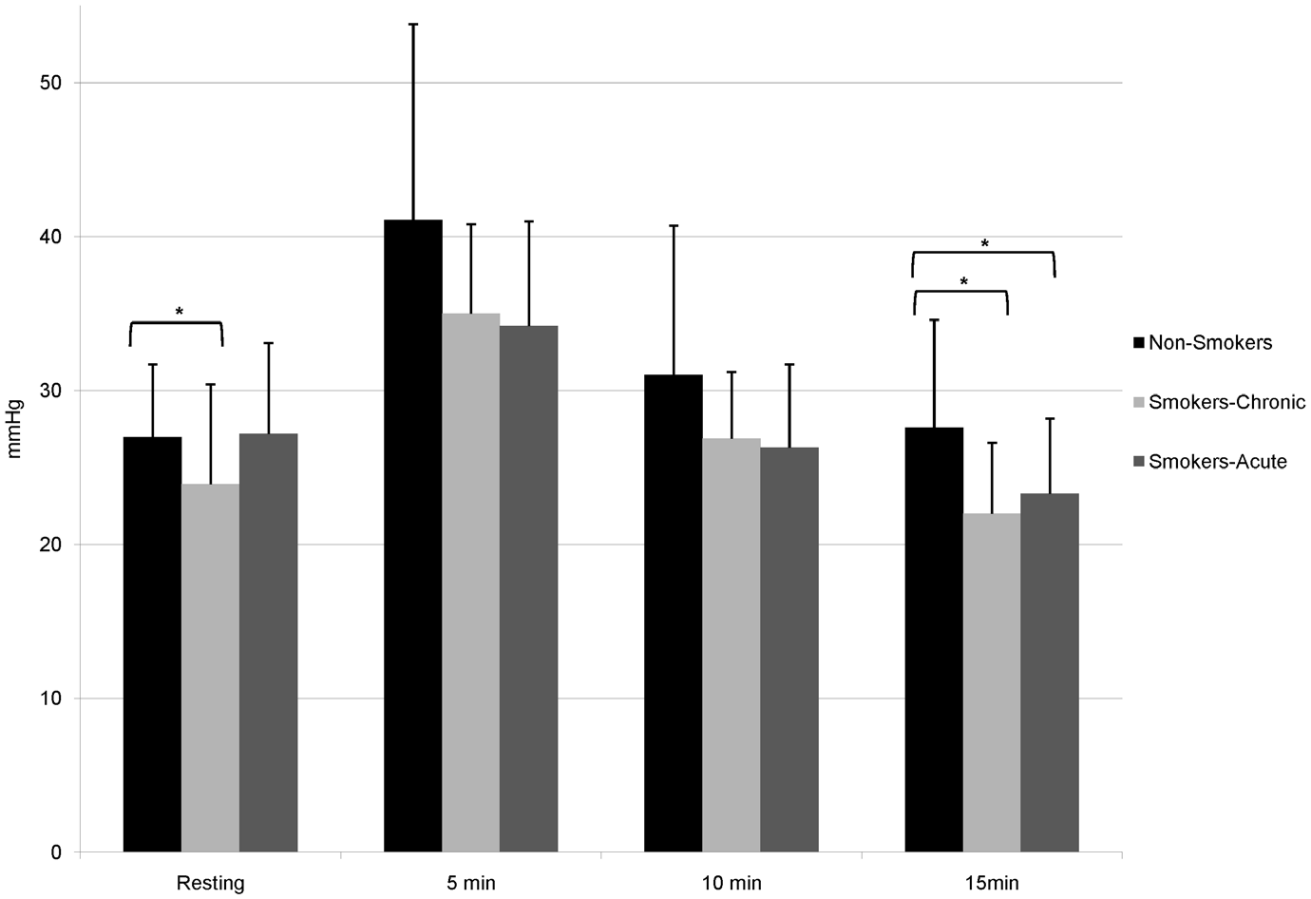
Aortic Pulse Pressure at Rest and Post-Exercise. Aortic pulse pressure at rest and after exercise in non-smokers and smokers in the chronic and acute conditions. *P<0.05 after adjustment.

**Figure 2:**
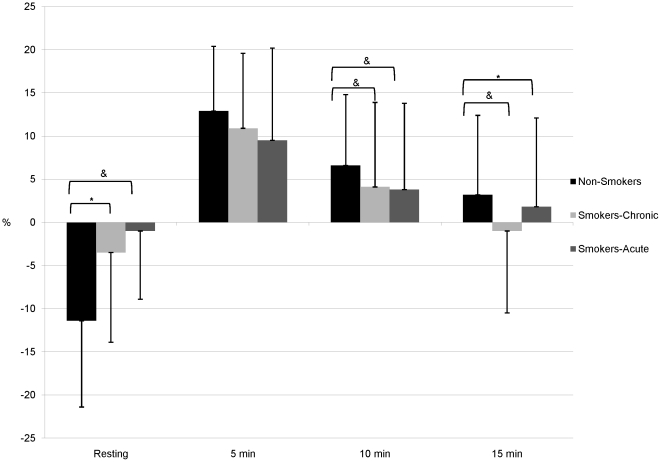
Augmentation Index at Rest and Post-Exercise. Augmentation index at rest and after exercise in non-smokers and smokers in the chronic and acute conditions. *P<0.05 after adjustment. ^&^P<0.01 after adjustment.

**Figure 3:**
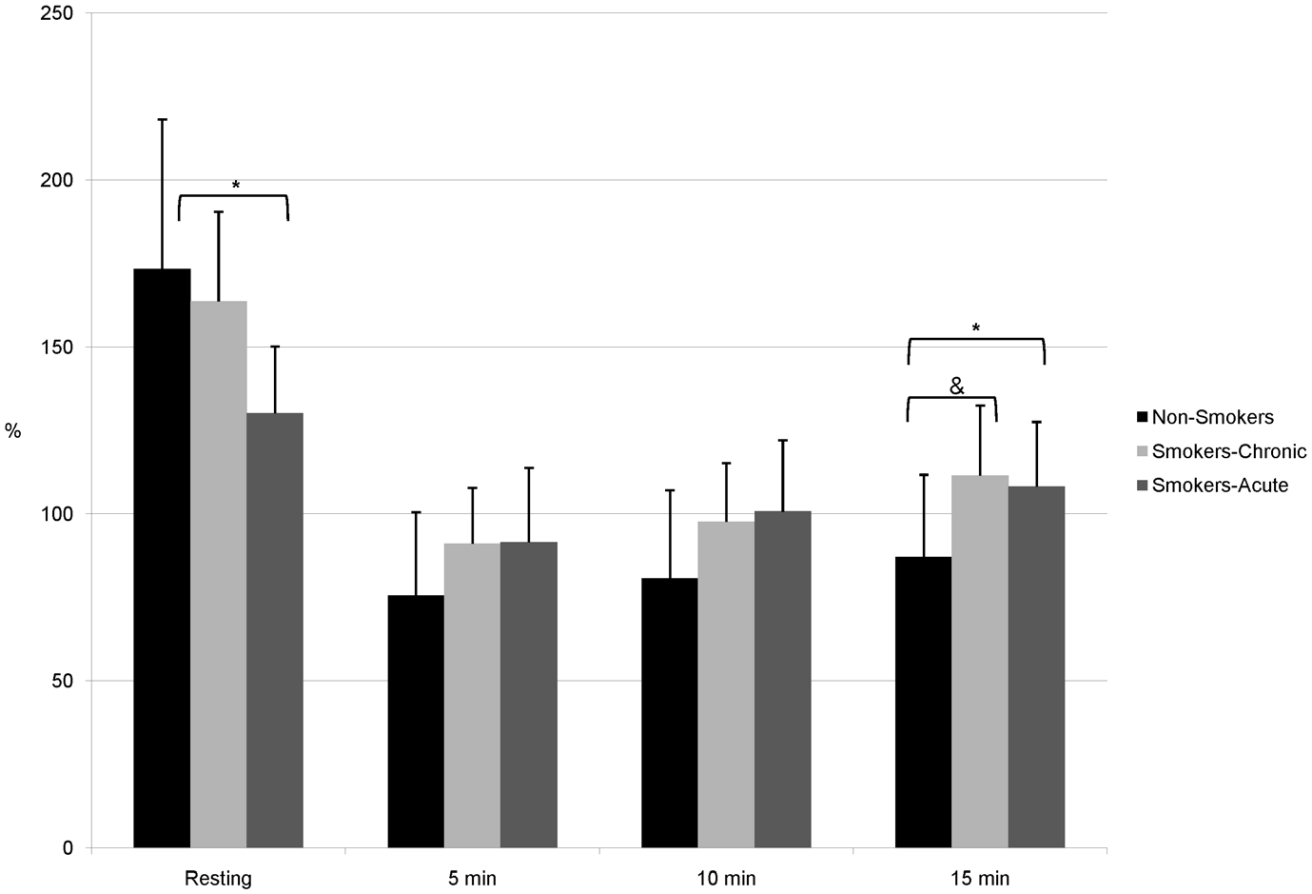
Subendocardial Viability Ratio at Rest and Post-Exercise. Subendocardial viability ratio at rest and after exercise in non-smokers and smokers in the chronic and acute conditions. *P<0.05 after adjustment. ^&^P<0.01 after adjustment.

Participant demographic information is shown in Table 1. In comparison to non-smokers, smokers were slightly older (26.0±6.7 vs. 23.1±5.4 years, P = 0.01). There was no significant difference between groups with respect to BMI or waist:hip ratio. Smokers reported smoking 10 cigarettes per day with a 2.9 pack-year history. Tables 2, 3, and 4 contain arterial stiffness and hemodynamic parameters at rest and post-exercise in non-smokers, smokers in the chronic condition, and smokers in the acute condition. Table 5 contains peak changes (pre-post exercise) and Table 6 contains AUC data and exercise parameters. Figures 1, 2, and 3 illustrate the aortic PP, AIx75, and SEVR, respectively in all 3 groups. Maximum heart rate and exercise time were significantly higher in non-smokers compared to smokers under both conditions (P<0.0001 for all). However, in smokers maximum heart rate did not differ significantly between chronic and acute conditions, while exercise time was significantly greater in the chronic condition (P<0.0001).

**Table 1:** Baseline Participant Characteristics.

	Smokers(n = 24)	Non-smokers(n = 53)	P Value
Age (years)	26.0±6.7	23.1±5.4	0.01
BMI (kg/m^2^)	21.3±4.2	22.3±2.2	NS
Waist:Hip Circumference	0.95±0.06	0.94±0.04	NS
Pack-years*	2.9 [1.0–5.6]	0	<0.0001
Cigarettes/day*	10 [4.7–12.0]	0	<0.0001

BMI, body mass index.

All values are mean±standard deviation except where values were not normally distributed. P values are unadjusted.

*presented as median values [interquartile range].

**Table 2:** Resting and Post-Exercise Vessel Hemodynamic Parameters - Non-Smokers vs. Chronic Smoking.

							P Values				
		Resting	2 min	5 min	10 min	15 min	Resting	2 min	5 min	10 min	15 min
HR	Non-S	62.6±8.0	111.7±15.3&	100.6±9.5&	97.7±9.8&	95.5±9.6&	NS	NS	0.01	0.02	0.02
	CS	61.7±8.0	104.8±15.0&	91.2±10.1&	88.3±10.5&	86.2±10.6&					
Aortic PP	Non-S	27.0±4.7	−	41.1±12.7&	31.0±9.7&	27.6±7.0	0.04	−	NS*	NS*	0.02
	CS	23.9±6.5	−	35.0±5.8&	26.9±4.3&	22.0±4.6&					
MAP	Non-S	82.2±8.9	94.1±12.5&	81.4±10.7	80.3±8.9	81.7±8.9	NS	NS	NS	0.01	0.03
	CS	83.2±8.4	94.2±10.8&	84.2±9.5	85.6±7.6	85.6±7.8					
cfPWV	Non-S	6.0±0.7	9.0±2.0&	6.6±1.3&	6.1±0.9	6.1±0.9	NS	NS	NS	NS*	NS*
	CS	6.3±1.0	8.8±1.7&	7.1±1.4&	6.7±1.0&	6.7±1.1&					
AIx75	Non-S	−11.4±10.0	−	12.9±7.5&	6.6±8.2&	3.2±9.2&	0.04	−	NS	0.01	0.003
	CS	−3.5±10.4	−	10.9±8.7&	4.1±9.8&	−1.0±9.5					
SEVR	Non-S	173.4±44.8	−	75.6±24.9&	80.7±26.4&	87.0±24.8&	NS	−	NS*	NS*	0.003
	CS	163.7±26.8	−	91.1±16.7&	97.8±17.4&	111.6±20.9&					

HR, heart rate (beats per minute); Non-S, non-smokers; CS, smokers-chronic smoking condition; PP, pulse pressure (mmHg); MAP, mean arterial pressure (mmHg); cfPWV, carotid-femoral pulse wave velocity (m/s); AIx75, augmentation index adjusted to heart rate of 75 beats per minute (%); SEVR, subendocardial viability ratio (%)

All values are mean±standard deviation.

* P<0.05 before adjustment.

& P<0.05 against resting value (i.e. this parameter has not recovered to resting levels).

Resting HR, cfPWV, AIx75, and SEVR are adjusted for age and resting MAP. Post-exercise, these variables are adjusted for age, exercise time, resting MAP, and the corresponding resting parameter.

Resting aortic PP and MAP were adjusted for age. Post-exercise, these variables were adjusted for age, exercise time, and the corresponding resting parameter.

**Table 3:** Resting and Post-Exercise Vessel Hemodynamic Parameters - Non-Smokers vs. Acute Smoking.

							P Values				
		Resting	2 min	5 min	10 min	15 min	Resting	2 min	5 min	10 min	15 min
HR	Non-S	62.6±8.0	111.7±15.3&	100.6±9.5&	97.7±9.8&	95.5±9.6&	<0.0001	0.05	0.0009	0.0003	0.0001
	AS	74.1±8.7	108.8±13.1&	91.3±13.0&	89.8±7.8&	88.8±7.7&					
Aortic PP	Non-S	27.0±4.7	−	41.1±12.7&	31.0±9.7&	27.6±7.0	NS	−	NS	NS*	0.05
	AS	27.2±5.9	−	34.2±6.8&	26.3±5.4	23.3±4.9&					
MAP	Non-S	82.2±8.9	94.1±12.5&	81.4±10.7	80.3±8.9	81.7±8.9	0.006	NS	0.07	NS*	0.06
	AS	89.1±7.1	93.9±9.7&	85.4±8.9&	87.1±7.6	89.5±7.1					
cfPWV	Non-S	6.0±0.7	9.0±2.0&	6.6±1.3&	6.1±0.9	6.1±0.9	NS*	NS	NS	NS*	NS*
	AS	6.7±1.0	9.3±2.5&	7.1±1.3&	6.8±1.2	6.6±0.9					
AIx75	Non-S	−11.4±10.0	−	12.9±7.5&	6.6±8.2&	3.2±9.2&	0.006	−	NS	0.01	0.04
	AS	−1.0±7.9	−	9.5±10.7&	3.8±10.0&	1.8±10.3					
SEVR	Non-S	173.4±44.8	−	75.6±24.9&	80.7±26.4&	87.0±24.8&	<0.0001	−	NS*	NS*	0.01
	AS	130.2±20.0	−	91.5±22.2&	100.7±21.4&	108.2±19.3&					

HR, heart rate (beats per minute); Non-S, non-smokers; AS, smokers-acute condition; PP, pulse pressure (mmHg); MAP, mean arterial pressure (mmHg); cfPWV, carotid-femoral pulse wave velocity (m/s); AIx75, augmentation index adjusted to heart rate of 75 beats per minute (%); SEVR, subendocardial viability ratio (%).

All values are mean±standard deviation.

* P<0.05 before adjustment.

& P<0.05 against resting value (i.e. this parameter has not recovered to resting levels).

Resting HR, cfPWV, AIx75, and SEVR are adjusted for age and resting MAP. Post-exercise, these variables are adjusted for age, exercise time, resting MAP, and the corresponding resting parameter.

Resting aortic PP and MAP were adjusted for age. Post-exercise, these variables were adjusted for age, exercise time, and the corresponding resting parameter.

**Table 4:** Resting and Post-Exercise Vessel Hemodynamic Parameters – Chronic Smoking vs. Acute Smoking.

		Smokers-Chronic					P Values				
		Resting	2 min	5 min	10 min	15 min	Resting	2 min	5 min	10 min	15 min
HR	CS	61.7±8.0	104.8±15.0&	91.2±10.1&	88.3±10.5&	86.2±10.6&	<0.0001	NS	NS	NS	NS
	AS	74.1±8.7	108.8±13.1&	91.3±13.0&	89.8±7.8&	88.8±7.7&					
Aortic PP	CS	23.9±6.5	−	35.0±5.8&	26.9±4.3&	22.0±4.6&	NS*	−	NS	NS	NS
	AS	27.2±5.9	−	34.2±6.8&	26.3±5.4	23.3±4.9&					
MAP	CS	83.2±8.4	94.2±10.8&	84.2±9.5	85.6±7.6	85.6±7.8	NS	NS	NS	NS	NS*
	AS	89.1±7.1	93.9±9.7&	85.4±8.9&	87.1±7.6	89.5±7.1					
cfPWV	CS	6.3±1.0	8.8±1.7&	7.1±1.4&	6.7±1.0&	6.7±1.1&	NS*	NS	NS	NS	NS
	AS	6.7±1.0	9.3±2.5&	7.1±1.3&	6.8±1.2	6.6±0.9					
AIx75	CS	−3.5±10.4	−	10.9±8.7&	4.1±9.8&	−1.0±9.5	NS*	−	NS	NS	NS
	AS	−1.0±7.9	−	9.5±10.7&	3.8±10.0&	1.8±10.3					
SEVR	CS	163.7±26.8	−	91.1±16.7&	97.8±17.4&	111.6±20.9&	0.03	−	NS	NS	NS
	AS	130.2±20.0	−	91.5±22.2&	100.7±21.4&	108.2±19.3&					

HR, heart rate (beats per minute); CS, smokers-chronic condition; AS, smokers-acute condition; PP, pulse pressure (mmHg); MAP, mean arterial pressure (mmHg); cfPWV, carotid-femoral pulse wave velocity (m/s); AIx75, augmentation index adjusted to heart rate of 75 beats per minute (%); SEVR, subendocardial viability ratio (%).

All values are mean±standard deviation.

* P<0.05 before adjustment.

& P<0.05 against resting value (i.e. this parameter has not recovered to resting levels).

Resting HR, cfPWV, AIx75, and SEVR are adjusted for resting MAP. Post-exercise, these variables are adjusted for exercise time and the corresponding resting parameter.

Resting aortic PP and MAP were unadjusted. Post-exercise, these variables were adjusted for exercise time and the corresponding resting parameter.

**Table 5:** Peak Pre-Post Exercise Changes.

	Non-smoker (1)	Smokers-Chronic (2)	Smoker-Acute (3)	P values		
				1 vs. 2	1 vs. 3	2 vs. 3
HR	49.1±14.7	43.0±12.8	34.7±13.1	NS	0.05	NS*
Aortic PP	13.9±11.4	10.0±6.7	6.9±8.7	NS	NS*	NS
MAP	11.4±10.6	11.0±10.0	4.8±9.7	NS	NS*	NS*
cfPWV	3.0±1.9	2.4±1.2	2.5±1.8	NS	NS	NS
AIx75	24.5±11.4	14.4±10.1	10.5±10.2	NS*	NS*	NS*
SEVR	−98.1±50.7	−72.6±22.4	−38.6±24.3	NS*	NS*	NS*

HR, heart rate (beats per minute); PP, pulse pressure (mmHg); MAP, mean arterial pressure (mmHg); cfPWV, carotid-femoral pulse wave velocity (m/s); AIx75, augmentation index adjusted to heart rate of 75 beats per minute (%); SEVR, subendocardial viability ratio (%).

All values are mean±standard deviation.

* P<0.05 before adjustment.

HR, cfPWV, AIx75, and SEVR are adjusted for age, exercise time, resting MAP, and the corresponding resting parameter between non-smokers and the two smoking groups (1 vs. 2 and 1 vs. 3). Repeated measures between Chronic and Acute smoking groups are adjusted for exercise time and resting MAP (2 vs. 3).

Aortic PP and MAP were adjusted for age, exercise time, and the corresponding resting parameter. Post-exercise, these variables were adjusted for age, exercise time, and the corresponding resting parameter between non-smokers and the two smoking groups. Repeated measures between Chronic and Acute smoking groups are adjusted for exercise time and resting MAP.

**Table 6:** Exercise Parameters and Area Under the Curve.

	Non-smokers (1)	Smokers - Chronic (2)	Smokers – Acute (3)	P values		
				1 vs. 2	1 vs. 3	2 vs. 3
Maximum HR (bpm)	195.0±9.0	185.7±9.3	184.6±10.2	<0.0001	<0.0001	NS
Maximum Exercise Time (mins)	16.18±1.6	14.5±1.3	14.1±1.4	<0.0001	<0.0001	<0.0001
MAP AUC	−11.2±73.3	19.7±46.1	−17.8±52.4	0.06	NS	NS
cfPWV AUC	7.8±9.6	9.3±8.2	5.9±7.3	NS	NS	NS
AIx75 AUC	191.3±100.4	75.7±86.7	58.1±81.7	0.01	0.006	NS*
SEVR AUC	−884.1±297.1	−662.1±205.2	−339.3±177.9	0.07&	<0.0001	0.01

HR, heart rate; MAP, mean arterial pressure; cfPWV, carotid-femoral pulse wave velocity; AIx75, augmentation index adjusted to heart rate of 75 beats per minute; SEVR, subendocardial viability ratio.

Values represented as mean±SD.

* P<0.05 unadjusted.

& P<0.01 unadjusted.

P values for 1 vs. 2 and 1 vs. 3, except MAP, are adjusted for age, resting MAP, and exercise time. MAP is adjusted for age and exercise time. All P values for 2 vs. 3, except MAP, are adjusted for resting MAP and exercise time. MAP is adjusted for exercise time.

### Study A – Smokers

#### Chronic Condition vs. Non-Smokers

Table 2 describes arterial stiffness and hemodynamic parameters at rest and post-exercise in smokers-chronic condition and non-smokers. Aortic PP was significantly higher in non-smokers at rest (P = 0.04) and 15 minutes (P = 0.02) post-exercise after adjustment and at 5 and 10 minutes before adjustment. cfPWV was not significantly different between groups at rest but was significantly higher in smokers 10 and 15 minutes post-exercise. However, this effect was lost after adjustment. Importantly, non-smoker cfPWV recovered faster; they had recovered to resting values by 10 minutes post-exercise while smokers' cfPWV remained significantly higher than their resting cfPWV throughout the entire post-exercise period. At rest AIx75 was significantly lower in non-smokers after adjustment, while at 10 and 15 minutes post-exercise AIx75 was significantly higher in non-smokers after adjustment. In addition, SEVR was not significantly different between groups at rest but was significantly lower in non-smokers post-exercise.

Peak changes (Table 5) were not significantly different between groups except for AIx75 and SEVR, which were significantly increased and decreased, respectively in non-smokers before adjustment.

### Study B1 – Smokers

#### Acute Condition vs. Non-Smokers

Table 3 describes arterial stiffness and hemodynamic parameters at rest and post-exercise in smokers in the acute condition and non-smokers. Aortic PP was significantly different between groups (higher in non-smokers) at 10 minutes (unadjusted) and 15 minutes post-exercise (adjusted). Acute smoking increased cfPWV compared to non-smokers at rest and at 10 and 15 minutes post-exercise. However, these effects were lost after adjustment. AIx75 was significantly higher after acute smoking at rest (P = 0.006). Although 10 and 15 minutes post-exercise there was a reversal of this trend and non-smokers had significantly increased AIx75. Indeed, non-smoker AIx75 had not recovered to resting levels by the end of the arterial stress test, while after acute smoking, smokers had recovered to resting levels by 15 minutes. SEVR was significantly higher in non-smokers at rest after adjustment but lower at 5 and 10 minutes post-exercise (before adjustment) and 15 minutes (with adjustment).

Peak changes in non-smokers for AIx75, SEVR, aortic PP, and MAP were all significantly greater compared to smokers - chronic condition before adjustment.

### Study B2 – Smokers

#### Chronic Condition vs. Smokers - Acute Condition

Acute smoking compared to chronic smoking significantly decreased SEVR and increased HR at rest after adjustment. Acute smoking also increased aortic PP, cfPWV, and AIx75 before adjustment at rest. There were no significant differences post-exercise except for significantly increased MAP after acute smoking before adjustment. Time to recovery for most parameters was similar in the chronic and the acute condition. However, cfPWV recovered earlier after acute smoking, likely due to the higher resting cfPWV compared to the chronic condition.

Peak change differences between chronic and acute smoking conditions for AIx75, SEVR, aortic PP, and MAP were significant before adjustment but were lost after adjustment.

### AUC Analyses

In order to simplify results we used AUC analyses and quantified the overall change in arterial stiffness and hemodynamic parameters over the whole time period of the arterial stress test (Table 6). AUC analysis showed AIx75 to be higher in non-smokers compared with smokers (chronic, P = 0.01) and after acute smoking (P = 0.006) after adjustment. Non-smokers had a significantly lower SEVR AUC (more negative, greater decrease in SEVR after exercise) compared with the chronic and acute conditions. However, adjusting reduced this to a trend in smokers-chronic condition (P = 0.07), while it remained significant between smokers-acute condition and non-smokers (P<0.0001). After adjustment, SEVR AUC was significantly higher in the acute condition compared with the chronic condition (P = 0.01). MAP AUC was trending towards a significant increase in smokers-acute condition compared with non-smokers after adjustment (P = 0.06).

## Discussion

We do not spend our lives at rest and physical stresses are a common occurrence in the daily life of almost every individual. Therefore, to capture more accurate indicators of disease risk it may be important to move beyond arterial stiffness and vessel hemodynamic measurements at rest. In this study, we used a method to quantify vascular function (at rest and post-exercise) termed the ‘arterial stress test’. Our findings suggest that while higher arterial stiffness, aortic PP and lower SEVR at rest is considered ‘detrimental’, this may not be the case for post-exercise.

We compared arterial stiffness and SEVR at rest and post-exercise between healthy, light smokers after 12h abstinence from smoking (Study A – chronic condition) and immediately after smoking 1 standardized cigarette (Study B – acute condition) and non-smokers using the arterial stress test. We were able to uncover extremely interesting differences between smokers and non-smokers with respect to SEVR, AIx75, and aortic PP. At rest, SEVR (indicator of O_2_ supply/demand) was significantly decreased after the acute smoking condition compared with non-smokers and the chronic condition. At several time points post-exercise the SEVR was significantly different between groups and there was a stepwise increase in SEVR AUC from non-smokers (lowest) to smokers-chronic condition, and smokers-acute condition (highest). This is interesting as this difference in SEVR between non-smokers and smokers-chronic condition was significant only after exercise (and not at rest) indicating that the greatest decrease in O_2_ supply/demand after physical stress was in non-smokers. This may appear counter-intuitive as insufficient cardiac perfusion for a given workload can lead to ischemia. However, SEVR must reach approximately 50% before there is considered to be cardiac ischemia[6]. It is likely that the non-smokers were simply able to stress their cardiovascular system more to achieve higher workloads (longer exercise time and higher level in the Bruce protocol) and therefore, bring themselves closer to the point of ischemia (peak SEVR at 5 minutes post-exercise was 75.6±24.9%) but not actually become ischemic. It is also of note that acute smoking appeared to blunt the effect of physical stress on the decrease in SEVR (smaller decrease in AUC). However, this may be due to the fact that at rest after acute smoking, SEVR was already reduced. In fact, smokers-chronic condition and smokers-acute condition reached similar peak SEVR at 5 minutes post-exercise (91.1±16.7% and 91.5±22.2%, respectively). However, this was achieved in a shorter exercise time in smokers-acute condition suggesting that acute smoking causes a decrease in the maximum attainable workload.

Aortic PP was found to be significantly higher in non-smokers at rest and post-exercise compared with chronic smokers while these differences were only noted post-exercise when compared to the acute condition. PP can be considered a surrogate for stroke volume[35], [36] and therefore, these differences are likely due to the increased workloads achieved by non-smokers. Indeed non-smokers were able to exercise for longer and at a higher stage in the Bruce protocol. Non-smoker mean maximum exercise time falls into stage 5, 5.0 miles/hour with 18% incline compared to both smoking conditions that fall into stage 4, 4.2 miles/hour with 16% incline. These results are in line with a previous study by Sharman et al. that found PP amplification during exercise is reduced with age and hypercholesterolemia[37].

Similar to SEVR, AIx75 was significantly elevated in the chronic and acute conditions compared with non-smokers at rest; however, after physical stress AIx75 was higher in non-smokers compared with smokers under both conditions. These findings could be a result of non-smokers achieving a higher heart rate or workload. However, AIx75 is adjusted to a heart rate 75 beats per minute and this finding remained significant after adjusting for exercise time.

Smoking causes endothelial dysfunction, increased production and release of endothelin-1 (ET-1), increased inflammation, decreased kidney function, insulin resistance, alterations in lipid metabolism, and increased oxidative stress, which reduces both production and bioavailability of nitric oxide (NO), and directly damages endothelial cells[38]–[42]. Furthermore, acute exercise causes a redistribution of blood flow to the working muscles caused by vasodilation of working muscles and vasoconstriction in other areas such as the splanchnic circulation[43], [44]. Exercise has been shown to increase ET-1 and NO release which play roles in blood redistribution during exercise[45]–[49]. Moreover, animal studies have shown that during exercise, local ET-1 production increases in the splanchnic circulation and decreases in the coronary circulation, and NO production increases in the coronary circulation[50]–[52]. Therefore, it is possible that after acute exercise in non-smokers, local production of ET-1 in the splanchnic and coronary circulations could be greater and lower than smokers, respectively; this could hold true even if total circulating ET-1 increases to a greater extent in smokers after exercise. This differential ET-1 release between vascular beds combined with greater NO release in the coronary circulation in non-smokers would allow non-smokers to achieve higher workloads bringing the heart closer to ischemia, explaining the lower SEVR post-exercise. In addition, this may also explain the increase in AIx75 seen post-exercise; greater vasoconstriction of the arteries to the gut in non-smokers would increase the number and magnitude of wave reflections in the aorta and abdominal aorta. Our findings at rest in smokers and non-smokers are in accordance with other studies that have also been performed at rest and have found that increased AIx is associated with CV risk and low SEVR to be associated with coronary artery disease, decreased coronary flow reserve in patients with healthy coronary arteries, severity of type 1 and type 2 diabetes, decreased renal function, and a history of smoking[6]–[12].

To our knowledge, no other studies compared these parameters in smokers and non-smokers after exercise, where different mechanisms could be implicated. After exercise, lower SEVR and higher AIx and aortic PP may simply represent a beneficial physiological response or a greater ‘vascular reserve’ that increases the ability to perform at higher workloads. Future studies will need to address the exact mechanisms behind these responses.

We also have found that acute smoking increased cfPWV at rest, but this effect was lost after adjusting for blood pressure. Chronic smoking was found to not have a significant effect on cfPWV at rest (chronic smoking condition vs. non-smokers). This is in line with a recent study that determined reference values for cfPWV[53]; they found no significant difference in cfPWV between middle aged smokers and non-smokers after adjusting for blood pressure. While there were no significant differences post-exercise in cfPWV between groups after adjusting but we did find that non-smokers had lower cfPWV before adjustment and a greater rate of recovery of cfPWV. Non-smokers returned to resting levels 10 minutes post-exercise while smokers-chronic condition were not recovered by the end of the arterial stress test. Smokers-acute condition did recover to resting levels before the arterial stress test was completed; however, this is due to their increased resting cfPWV. By enrolling only young, healthy, light smokers (median pack-years of 2.9) in our study we assessed the scenario least likely to cause changes in arterial stiffness or vessel hemodynamics (i.e. minimal vascular damage). Therefore, the arterial stress test was able to elicit changes in cfPWV in subject with little vascular damage due to chronic smoking. To our knowledge this is the first study to capture the acute response of the vascular system immediately after maximal physical stress by measuring cfPWV (the ‘gold standard’ measure of arterial stiffness), and therefore, provides valuable information that can be used to help design future studies.

Taken together, our findings suggest that the new method we have used, the arterial stress test, may in the future offer novel information to better estimate clinical risk and monitor health status over time, which may be applied to patient groups with minimal vascular damage. Using the arterial stress test we were able to elicit evidence of vascular impairment in young healthy light smokers at an earlier stage, before vascular damage was detectable at rest. Earlier detection of vascular compromise may offer opportunities to intervene and attenuate or reverse vascular dysfunction and cardiovascular risk at an earlier stage. The arterial stress test may offer the opportunity to move beyond our current methods of addressing smoking as a binary risk factor (present/absent), and vascular damage can be quantified directly rather than inferred indirectly through exposure (e.g. pack-years). Enhanced risk stratification would allow clinicians to identify those individuals at higher risk (independently of their smoking status: current, never, former), monitor them closely, and treat them aggressively when indicated.

There are limitations in this study. An inherent limitation is the relatively small sample size (n = 77). Yet, despite this we were able to show significant differences in vessel hemodynamic parameters between smokers and non-smokers at rest and post-exercise; this provides evidence for the arterial stress test being an effective method to elicit even subtle differences in arterial stiffness. Due to the technical limitations of applanation tonometry, we were not able to measure arterial stiffness throughout exercise, only immediately post-exercise. Furthermore, since the time limitations for measurements post-exercise were strict, we did not perform the PWA analysis at 2 minutes. We recruited consecutive eligible subjects and therefore, we did not age-match our subjects, instead we adjusted for age. We included only men in order to avoid potential sex effects on vascular response. The ratio of non-smokers to smokers is 2∶1; this resulted in increased power compared to a 1∶1 ratio with n = 24 in each group[54]. Blood tests to measure CV risk factors were not available. Furthermore, this study is hypothesis generating and the natural first step before validating the arterial stress test and the ‘vascular reserve’ concepts in future larger studies with target organ damage and clinical events as end points. We did not measure VO_2max_ to confirm that the subjects reached their maximal exercise capacity. However, we used three well established criteria, as mentioned above[32]. We also did not have a non-smoker group perform the acute smoking intervention. However, this was deemed unethical by the McGill University Health Centre Research Ethics Board.

### Conclusions

We have assessed arterial stiffness and hemodynamic parameters before and after exercise using a novel protocol (the arterial stress test). Young, healthy, light smokers exhibited blunted decreases in SEVR and blunted increases in AIx75 and aortic PP after acute physical stress compared with non-smokers. This shows that even in this relatively healthy population there are impairments in the ability of the arteries to respond to physical stress before changes are noted at rest, suggesting that it is possible to identify the development of vascular damage at an earlier stage than current tools allow. If this ability to respond to increased demands of physical stress in non-smokers can be considered a ‘vascular reserve’ remains to be confirmed. Our findings also indicate that an increase in arterial stiffness or a greater decrease in SEVR after physical stress may play a beneficial role in the physiological response to exercise, providing further support for the need to assess ‘vascular reserve’. This hypothesis will need to be investigated in future studies. The arterial stress test may be used in the future to better stratify individual risk, intervene earlier and attenuate the high rates of morbidity and mortality associated with smoking and cardiovascular disease.
